# Pathway of FeEDTA transformation and its impact on performance of NO_*x*_ removal in a chemical absorption-biological reduction integrated process

**DOI:** 10.1038/srep18876

**Published:** 2016-01-08

**Authors:** Wei Li, Jingkai Zhao, Lei Zhang, Yinfeng Xia, Nan Liu, Sujing Li, Shihan Zhang

**Affiliations:** 1 Key Laboratory of Biomass Chemical Engineering of Ministry of Education, Institute of Industrial Ecology and Environment, College of Chemical and Biological Engineering, Zhejiang University (Yuquan Campus), Hangzhou, 310027, China; 2 Institute of Environmental Engineering, Zhejiang University (Zijingang Campus), Hangzhou, 310058, China; 3 Zhejiang Industrial Environmental Protection Design & Research Institute Co., Ltd., Hangzhou, 310035, China

## Abstract

A novel chemical absorption-biological reduction (CABR) integrated process, employing ferrous ethylenediaminetetraacetate (Fe(II)EDTA) as a solvent, is deemed as a potential option for NO_*x*_ removal from the flue gas. Previous work showed that the Fe(II)EDTA concentration was critical for the NO_*x*_ removal in the CABR process. In this work, the pathway of FeEDTA (Fe(III)/Fe(II)-EDTA) transformation was investigated to assess its impact on the NO_*x*_ removal in a biofilter. Experimental results revealed that the FeEDTA transformation involved iron precipitation and EDTA degradation. X-ray photoelectron spectroscopy analysis confirmed the iron was precipitated in the form of Fe(OH)_3_. The iron mass balance analysis showed 44.2% of the added iron was precipitated. The EDTA degradation facilitated the iron precipitation. Besides chemical oxidation, EDTA biodegradation occurred in the biofilter. The addition of extra EDTA helped recover the iron from the precipitation. The transformation of FeEDTA did not retard the NO removal. In addition, EDTA rather than the iron concentration determined the NO removal efficiency.

Fine particulate matter with an aerodynamic equivalent diameter lower than 2.5 micron (PM_2.5_) causes critical ecological and environmental issues^1^,^2^. Nitrogen oxides (NO_*x*_) are major precursors to form PM_2.5_^3^,^4^. NO_*x*_ can also result in other environmental issues such as acid rain, ozonosphere depletion, and urban ozone smoke. Therefore, the emission limitation of NO_*x*_ from thermal power stations, major source of NO_*x*_ emissions, is stringent.

To control NO_*x*_ emissions, various technologies, for instance, selective catalytic reduction (SCR), selective non-catalytic reduction (SNCR), absorption, and adsorption, have been developed in the past decades^5^,^6^,^7^. All these technologies cannot meet all the requirements, namely low cost, high efficiency, and non-secondary pollution. The biological technology is regarded as an effective and environmentally friendly approach^8^. However, its removal efficiency of NO_*x*_ is limited by NO mass transfer from gas phase to the liquid phase because of its high Henry’s constant^9^. Recently, an innovative chemical absorption-biological reduction (CABR) integrated system, adopting the advantages of both chemical absorption and biological treatment, has been developed^10^,^11^,^12^. In the CABR process, ferrous ethylenediaminetetraacetate (Fe(II)EDTA) is used as a solvent to absorb NO and hence enhance the NO mass transfer rate. The bound NO, Fe(II)EDTA-NO, is then biologically reduced to N_2_ by denitrifiers. Meanwhile, the byproduct Fe(III)EDTA, formed via the oxidation of Fe(II)EDTA by oxygen, is biologically reduced to Fe(II)EDTA by iron-reducing bacteria. The principle of the CABR process has been well documented in our previous work^13^.

It has been reported that the biological reduction rate of Fe(III)EDTA determines the performance of NO removal via the CABR process^14^. The reduction rate of Fe(III)EDTA was 5.17 × 10^−5^ mol m^−2^ min^−1^, which was only half of that of the bound NO^15^. Zhang *et al.*^16^ reported that during the operation of the CABR process, the total iron concentration in the recirculated solution dropped gradually in a biofilter. Based on the iron mass balance, the average iron loss in the biofilter was 0.83 M d^−1^ in the first 28 days in presence of 3% (v/v) oxygen^16^. van der Maas *et al.*^17^ also showed that 2 mM d^−1^ of EDTA degradation occurred during the long-term operation of NO removal in presence of 3.5–3.9% (v/v) oxygen. They also confirmed that the degradation of EDTA was due to the chemical oxidation by the oxygen in the scrubber rather than in the bioreactor since the dissolved oxygen could only be expected at the gas-liquid interface in the scrubber^17^. However, the information, how the iron loss and EDTA degradation impact the NO removal, was not available in the literature. Meanwhile, the iron transformation (loss) pathway and EDTA degradation mechanism, which can provide some insight to prevent the FeEDTA (Fe(III)/Fe(II)-EDTA) loss and thus reduce the CABR operation cost, remain unknown.

In this work, we aimed to figure out the FeEDTA transformation pathway and its influence on NO_*x*_ removal performance. Iron transformation and EDTA degradation were determined to identify the fate of FeEDTA in the CABR system with a long-term operation. Meanwhile, a relationship between the FeEDTA transformation and NO_*x*_ removal efficiency was also determined. This work may provide some insight on how to maintain the Fe(II)EDTA at a certain level and hence sustain the continuous NO_*x*_ removal as well reduce the operation cost for the practical application.

## Results and Discussion

### Iron concentration profile during the long-term operation for NO_*x*_ removal

**Figure 1:**
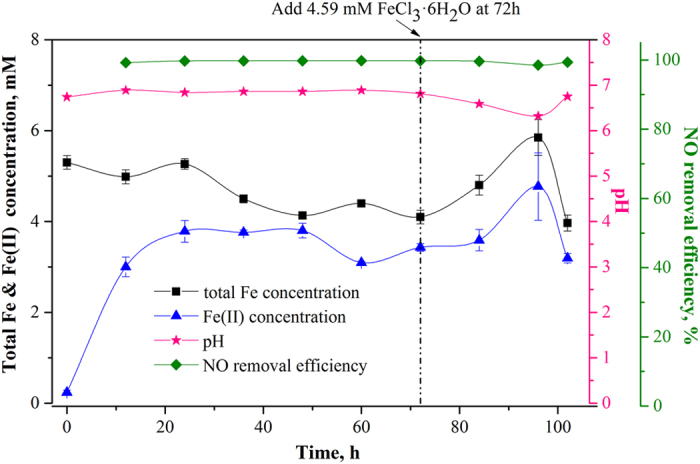
Profile of Fe(II), total iron, NO removal efficiency, and pH during the operation of CABR system. ([Fe(III)EDTA]_0_, 5 mM; CO_2_, 15% (v/v); O_2_, 3% (v/v); NO, 400 ppm; gas flow rate (*G*), 1 L min^−1^; circulative liquid flow rate (*V*_*L*_), 40 L h^−1^; *T*, 50 °C; pH, 6.8-7.0).

Figure 1 shows the profile of Fe(II) concentration, total iron concentration, NO removal efficiency and pH value during the 102 h operation of CABR system under the conditions of 5 mM FeEDTA, 3% O_2_ (v/v), and 400 ppm NO. Fe(II) concentration was the sum of the concentrations of Fe(II)EDTA and Fe(II)EDTA-NO. The new system used in this work reached steady-state within 8 hours (details can be seen in Figure S1, Supporting information). In the absence of oxygen, the maximum iron reducing rate in this biofilter was about 1.87 mM h^−1^ (Figure S1), which was almost twice of that reported in our previous study^18^, indicating the high activity of microbes applied in this work.

The total iron concentration showed a decreasing trend in the first 72 h, as shown in Fig. 1. The concentration of Fe(II) increased from 0 to 3.85 mM due to the reduction of Fe(III) and reached the plateau after 24 h. When additional Fe(III) (as the form of FeCl_3_∙6H_2_O) was supplied, Fe(II) concentration was fluctuant as the same pattern as the total Fe. Although the total iron gradually decreased from 20 to 48 h, the concentration of Fe(II) almost kept constant during the same timeframe, resulting in a constant NO removal efficiency (~99%). These results confirmed that the NO removal efficiency was influenced by Fe(II)EDTA concentration rather than the total iron concentration in the solvent^14^, because it was the former that complexed with NO.

### Pathway of iron transformation

In the solution, the distribution of iron is determined by the following equilibriums:


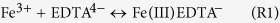



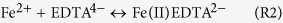



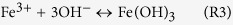



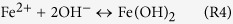



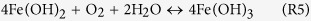


Based on the thermodynamic analysis (see details in the supporting information), it is possible to form Fe(OH)_3_ via the reaction R3 under the typical CABR conditions. To that end, the solids located in storage tank and packing materials, labelled as S-D and S-B respectively, were collected for XPS analysis. As depicted in Figure S2, the samples S-D and S-B contained Fe (2p), O (1s), N (1s), C (1s), and P (2p). Besides those elements, S-B also contained S (2p). The atomic ratios of different elements and their corresponding binding energies are listed in Table S1.

**Figure 2:**
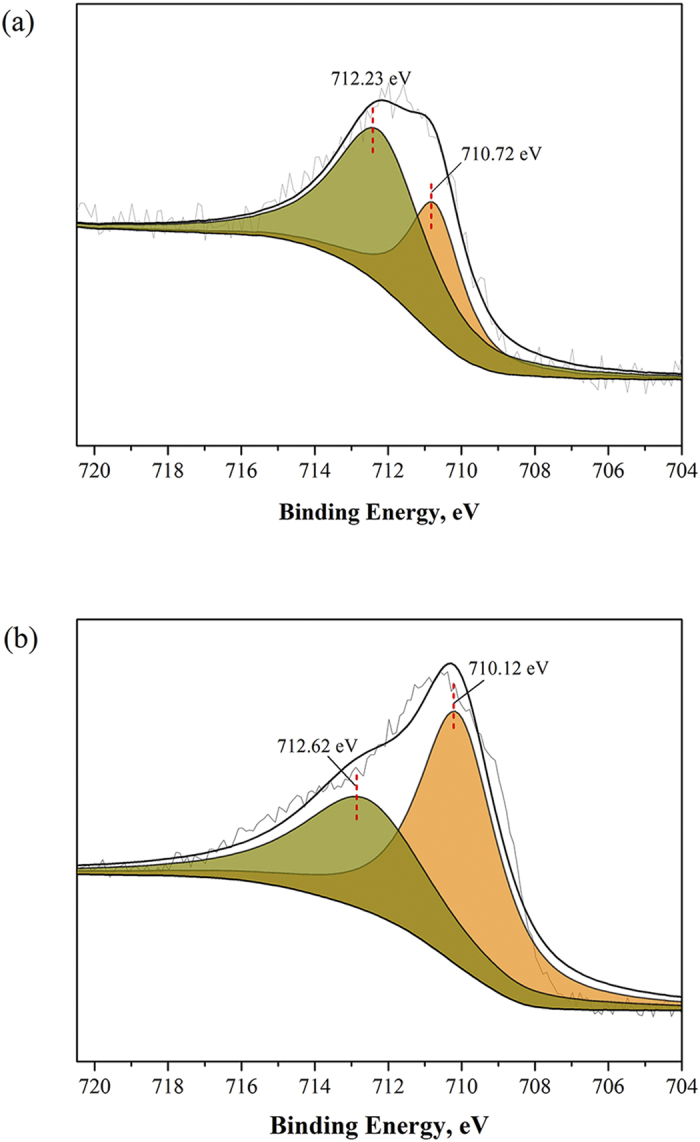
XPS spectra of Fe2p3/2 for the samples. (**a**) S-D and (**b**) S-B.

Figure 2 shows the XPS spectra of Fe2p3/2 for the samples S-D and S-B. The Fe2p3/2 spectra for S-D consisted of two peaks, one at the binding energy of 710.72eV and the other at 712.23eV. As reported^19^,^20^,^21^,^22^, the major peak located at 712.23eV corresponds to lattice Fe(III)-O in Fe_2_O_3_ (65.5%), and the peak located at 710.71eV is due to (O)Fe-OH species in Fe(OH)_3_ (34.5%). It should be noted that Fe_2_O_3_ can not be formed in the soltuion. The detected Fe_2_O_3_ was the product of the dehydration of Fe(OH)_3_ during the XPS pretreatment. The formation of Fe_2_O_3_ may also occur because of the aging of the Fe(OH)_3_ during the long-term operation, e.g. several month-operation. On the other hand, the peaks of the Fe2p3/2 spectra in S-B sample located at 712.62eV and 710.12eV, which were assigned for Fe(III)-O (41.78%) and (O)Fe-OH (58.22%)^19^,^20^,^21^,^22^. It should be noted that the solubility product of Fe(OH)_3_ is twelve magnitude order lower than that of Fe(OH)_2_^23^. Thus, in this system, Fe^3+ ^deposited prior to Fe^2+ ^. Overall, the iron loss in the CABR system occurred via the formation of Fe(OH)_3_. XPS results also confirmed that no FeS was formed during the operation of CABR process.

To quantify the amount of the iron loss during the long-term operation, a mass balance of iron was determined. As shown in Table 1, the iron inlet inluded the amount of iron added at time = 0 h and time = 72 h. The iron outlet was cataloged into: 1) iron retained in the solution, 2) iron precipitated in the storage tank, and 3) iron loss due to the sampling. The total difference between iron inlet (39.550 mmol) and outlet (35.762 mmol) was 3.788 mmol, accounting for 9.58% of the iron inlet. The imbalance of the iron in this work may be due to the iron precipitation in the pipeline and on the packing materials since the biofilter was washed using the natural basal medium rather than the acid solution. Amongst the iron outlet, the iron precipitation, regarded as main product of iron loss during the operation, accounted for 44.2% of the total iron inlet. Theoretically, the iron loss can be only 37% as determined by the thermodynamic analysis if no EDTA degradation occurs (see Supporting information), indicating that EDTA may be degraded in the CABR process and thus facilitates the iron precipitation.

### EDTA degradation facilitating iron precipitation

**Figure 3:**
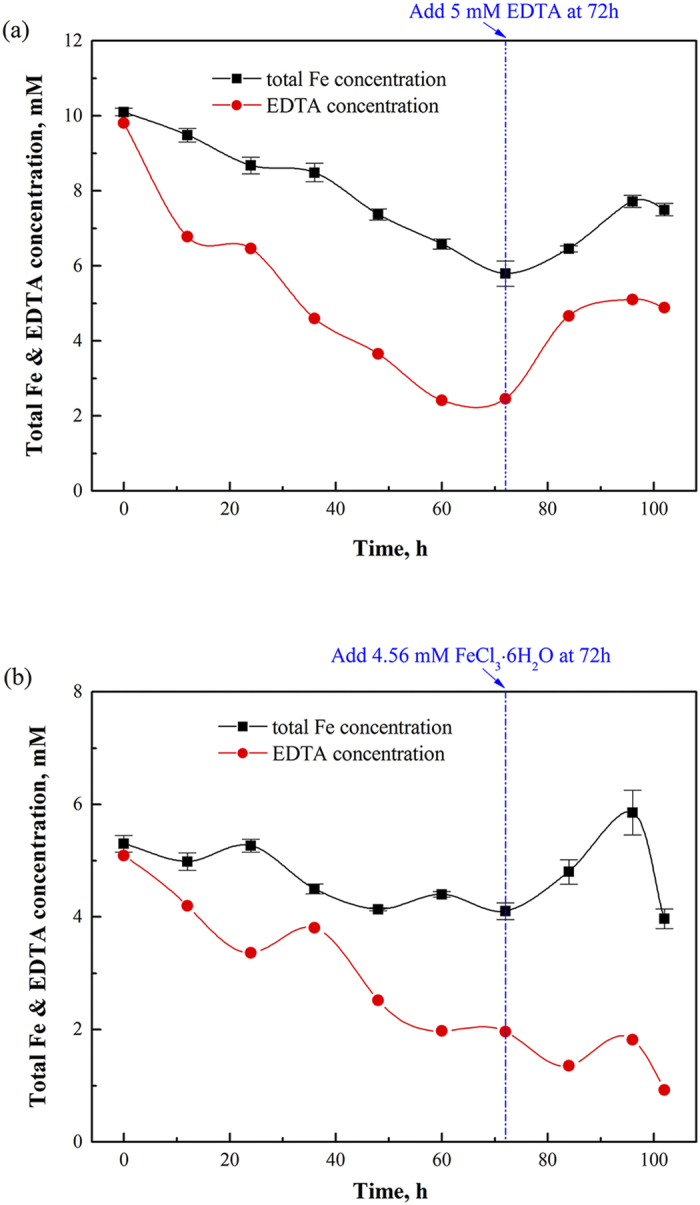
Profile of iron loss and EDTA degradation at various Fe(III)ETA concentrations. (**a**) [Fe(III)EDTA]_0_, 10 mM; (**b**) [Fe(III)EDTA]_0_, 5 mM. (O_2_, 3% (v/v); NO, 400 ppm; *G*, 1 L min^−1^; *V*_*L*_, 40 L h^−1^; *T*, 50 °C; pH, 6.8–7.0).

As shown in the Fig. 3(a), EDTA degradation occurred during the operation of the CABR process. The concentration of EDTA decreased from 10 to 2.46 mM during the 72 hours of operation. On the other hand, the dosage of extra 5 mM EDTA into the solution resulted in an increase of total iron concentration from 5.79 to 7.72 mM, indicating the iron precipitation can be recovered with the aid of EDTA. However, the supplement of Fe(III) could not mitigate the EDTA degradation, see Fig. 3(b). The degradation of EDTA accelerated the formation of Fe^3+^ ion in the solution as described in R1 and in turn enhanced the reaction of R3, resulting in an increase of Fe(OH)_3_ formation.

**Figure 4:**
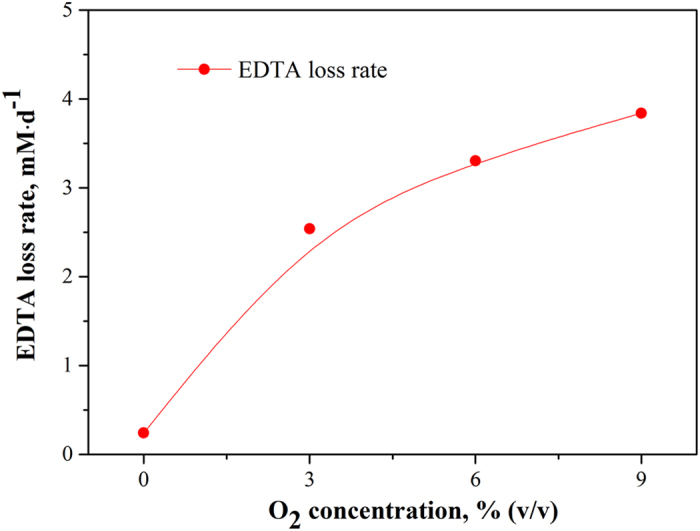
Relationship between O_2_ concentration and EDTA loss rate. ([Fe(III)EDTA]_0_, 10 mM; CO_2_, 15% (v/v); NO, 400 ppm; *G*, 1 L min^−1^; *V*_*L*_, 40 L h^−1^; *T*, 50 °C; pH, 6.8–7.0).

The average EDTA degradation rate in 72 hours was ~1.0 mM d^−1^ at 50 ^o^C and 5 mM FeEDTA in presence of 3% (v/v) oxygen (Fig. 3(b)). As the initial concentration of FeEDTA increased from 5 to 10 mM, the average EDTA degradation rate increased from 1.0 to 2.54 mM d^−1^, indicating the EDTA degradation rate enhanced as the initial FeEDTA concentration increased. Moreover, as depicted in Fig. 4, the average degradation rate of EDTA was also accelerated as the O_2_ concentrations increased. The average degradation rate during 72 h operation increased from 2.54 to 3.84 mM d^−1^ as the oxygen concentration in the feeding gas increased from 3 to 9% (v/v) in presence of 10 mM FeEDTA.

O_2_ played an important role in EDTA degradation as shown in Fig. 4. It has been reported that EDTA degradation can occur via chemical oxidation and biological degradation^17^,^24^,^25^. Chemical oxidation of EDTA took place in the biofilter because it was unavoidable in presence of O_2_. Radical formation occurred via iron-mediated Haber–Weiss reactions as follows^24^:


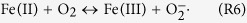



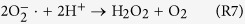



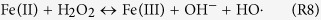


The EDTA degradation via chemical oxidation occurred in presence of the produced radicals. Furthermore, iron-chelate complexes were thought to be capable of forming strong oxidants during the autoxidation. The oxidant formed during autoxidation of Fe(II)EDTA was more active than a free hydroxyl radical, suggesting some type of hypervalent iron complex was formed^26^. As reported, the chemical oxidation of EDTA may induce formations of small-molecular organics, such as ethylenediamine triacetic acid, iminodiacetic acid, and acetic acid^27^,^28^.

**Figure 5:**
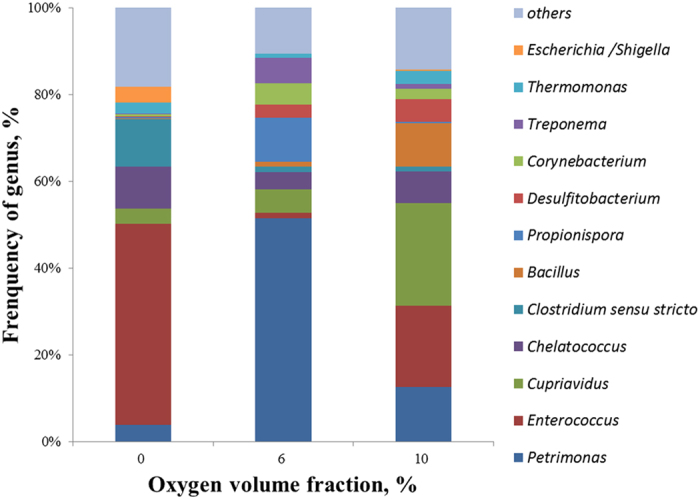
Microbial community distribution under different oxygen concentrations.

Besides chemical oxidation of EDTA, biological degradation of EDTA can also occur under aerobic condition^27^,^29^,^30^. In this work, the mixed culture for inoculation was enriched from the sewage sludge, while EDTA biodegradation was observed in the conventional wastewater treatment plant^29^,^31^. Therefore, it can be inferred that the biofilm in the biofilter had the ability of EDTA degradation. To confirm this speculation, the microbial community distributions under different oxygen concentrations were investigated. It has been confirmed that the bacteria *Bacillus* can degrade EDTA^32^,^33^. As shown in Fig. 5, the amount of genus *Bacillus* was increased from 0.23%, 1.15%, to 9.90% while O_2_ concentration was increased from 0%, 6%, to 10%. As a result, the biodegradation rate of EDTA was accelerated. On the other hand, some gram-negative, including an *Agrobacterium radiobacter* strain^34^, *Escherichia coli* BL21^35^, *Pseudomonas* sp. A1^36^, DSM 9103^30^,^37^,^38^ can use EDTA as nitrogen source. Thus, the gram-negative bacteria, such as *Escherichia/Shigella, Cupriavidus, Petrimonas,* and *Chelatococcus*, in the biofliter may also contribute to the EDTA degradation. Overall, both chemical oxidation and biological degradation were contributed to the EDTA degradation under the tested conditions in this work.

### Impact of FeEDTA transformation on NO_*x*_ removal performance

**Figure 6:**
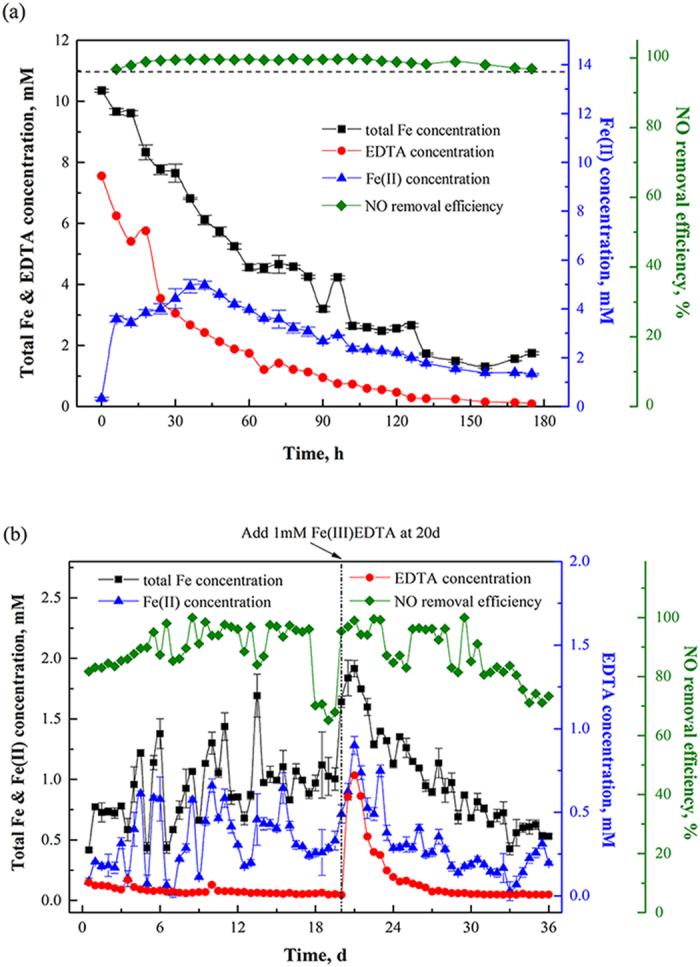
(**a**) Effect of iron loss and EDTA degradation on the NO removal efficiency (initial concentration of 10.35 mM total Fe and 7.55 mM EDTA); (**b**) Biofilter performance at low iron and EDTA concentration (initial concentration of 0.78 mM total Fe and 0.085 mM EDTA). (NO, 400 ppm; O_2_, 6% (v/v); *G*, 1 L min^−1^; *V*_*L*_, 40 L h^−1^; *T*, 50 °C; pH, 6.8–7.0).

To determine the impact of the FeEDTA transformation on the NO_*x*_ removal performance, the biofilter was operated for 180 h without adding extra EDTA and iron under the condition of ~10 mM total iron, ~7.55 mM EDTA, 6% (v/v) oxygen, and 50 ^o^C. As shown in Fig. 6(a), the NO removal efficiency kept above 96.8% even when the total Fe and EDTA concentration declined to around 1.75 and 0.09 mM, respectively, indicating the iron loss and EDTA degradation did not noticeably impact the NO removal efficiency and the CABR can maintain high NO removal efficiency even at low concentration of Fe(II)EDTA. Thus, dosage of extra FeEDTA is not necessary to maintain the NO removal efficiency in the practical application.

To identify the critical component (Fe(II) or EDTA) in the Fe(II)EDTA that determines the NO removal efficiency, the biofilter was operated at conditions of low concentration of iron and EDTA, e.g., 0.78 mM total iron and 0.085 mM EDTA with a feeding gas of 400 ppm NO and 6% (v/v) oxygen. In the first 17 days’ operation, see Fig. 6(b), NO removal efficiency of the CABR integrated system maintained above 80%. When the EDTA concentration was below 1.75 × 10^−2^ mM, the NO removal efficiency dropped sharply from 90% to 70%. On the other hand, in the first 17 days’ operation, the total iron amount was not noticeably changed which may be due to the thermodynamic equilibrium of iron reached in the biofilter. The system performance was recovered after adding another 1 mM Fe(III)EDTA into the system, proving that the drop in NO removal efficiency was due to the limited accession of the EDTA in the system. When the EDTA amount dropped below 1.75 × 10^−2^ mM again at days 33, the NO removal efficiency dropped to ~70%. Thus, to maintain high NO removal efficiency, it is critical to keep the concentration of EDTA above a certain level rather than the iron.

## Conclusions

The biofilter used in this work presented a high Fe(III)EDTA reduction rate compared with our previous work. XPS results confirmed that the pathway of iron loss was via formation of Fe(OH)_3_. Mass balance analysis of iron showed that 44.2% of iron precipitated during the 102 hours’ operation. The degradation of EDTA, which occurred via both chemical oxidation and biological degradation, induced the iron precipitation. The NO removal efficiency was not impacted by the FeEDTA transformation at the typical operation conditions. Moreover, it was EDTA rather than iron concentration that determined the NO removal efficiency. These results provided important information for the operation of the CABR process to achieve high NO removal efficiency and low operation cost in the practical application.

## Experimental

### Chemicals

Na_2_EDTA·2H_2_O (99%), FeSO_4_(NH_4_)_2_SO_4_·6H_2_O (99.5%), FeCl_3_·6H_2_O (99%), and D-glucose (99.5%) were acquired from Sinopharm Chemical Reagent Co. (Shanghai, China). NO (5% in N_2_, v/v), N_2_ (99.999%), CO_2_ (99.999%) and O_2_ (99.999%) were provided by Zhejiang Jingong Gas Co. (Hangzhou, China). All other chemicals are analytical-reagent grade, commercially available and used without further purification.

### Medium and organism

The basal medium consisted of following components (mg L^−1^): Glucose, 1000; KH_2_PO_4_, 300; MgCl_2_, 100; Na_2_SO_3_, 70; CaCl_2_, 20; trace elements, 2. The composition of the trace elements for the bacteria growth was as follows (mg L^−1^): MnCl_2_∙4H_2_O, 990; CuSO_4_∙5H_2_O, 250; CoCl_2_, 240; NiCl_2_∙6H_2_O, 190; ZnCl_2_, 100; H_3_BO_4_, 14.

The denitrifying and Fe(III)EDTA reducing bacteria used in this work were enriched from the activated sludge of denitrifying reactor in Hangzhou Qige sewage treatment plant by modifying the nutrient content of the culture medium. The enrichment cultures without further isolation were used for the inoculation of the biofilter. The details on the enrichment of those two types of bacteria can be found in our previous work^39^. It should be note that microorganisms applied in this work were two kinds of mixed strains with desired function.

### Microorganism acclimation in the biofilter

**Figure 7:**
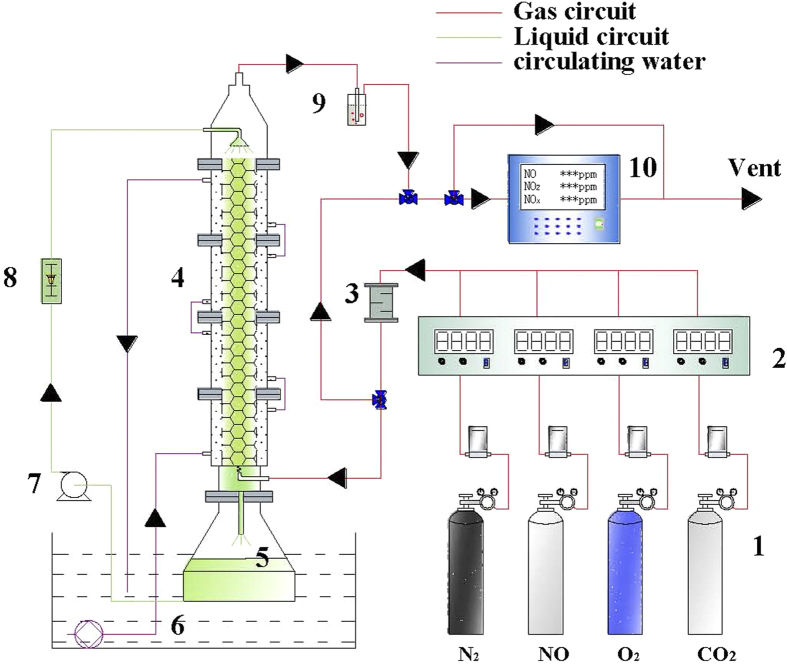
Schematic of the CABR system. 1. Gas cylinder; 2. Mass flow controller; 3. Gas mixing chamber; 4. Biofilter, 5. Solvent storage tank; 6. Thermostatic water bath; 7. Solvent recycle pump; 8. Liquid flow meter; 9. Cold trap; 10. NO_*x*_ analyzer.

Figure 7 shows the schematic of CABR integrated system. A biofilter with an inner diameter of 0.08m and an effective volume of 2 L was used for the microorganism acclimation. 1.5 L of polyvinyl chloride cross ring (Pengxiang xingfeng chemical packing Co., Ltd., China) with a specific surface area of 1200 m^2^ m^−3^ was packed into the biofilter. The temperature of the biofilter was controlled by a water jacket. Detailed description of this set-up can be found in our previous work^40^.

4 L of basal medium, containing 20 mM Fe(III)EDTA and 0.15 g DCW L^−1^ iron reducing bacteria with a pH value of 6.8, was used as initial solution for the inoculation of iron reducing bacteria. At the first period, 15% (v/v) CO_2_ and balanced N_2_ were fed into the biofilter at a flow rate of 1 L min^−1^. Meanwhile, 2 L of the circulated solution was replaced by fresh medium daily until the biofilm was visible on the packing. Once the Fe(III)EDTA reduction rate reached 1 mM h^−1^, the concentration of O_2_ in the feeding gas was gradually raised to 3% (v/v). After the inoculation of iron reducing bacteria, acclimation of Fe(II)EDTA-NO reducing bacteria was proceeded via the addition of 0.15 g DCW L^−1^ denitrifying bacteria under the conditions of 100–500 ppm NO and 3-6% (v/v) oxygen. A steady NO removal efficiency above 95% was used as an indicator of the completion of the inoculation of the denitrifying bacteria.

### Experimental procedures

After the acclimation of the biofilter, the performance of long-term NO removal was conducted. In a typical test, the biofilter was loaded with simulated flue gas containing NO (0–500ppm), O_2_ (0–12%, v/v), CO_2_ (15%, v/v) and balanced N_2_ under the conditions of a gas flow rate of 1 L min^−1^, a liquid flow rate of 40 L h^−11^, and 5–10 mM of Fe(III)EDTA. In order to explore the FeEDTA transformation, two main components of FeEDTA, iron and EDTA, were monitored during the operation of the biofilter. Meanwhile, the precipitations in the storage tank and on the packing materials were collected for iron analysis. To investigate the influence of the EDTA on the iron loss, 5 mM of extra EDTA was added into circulated solution after 72 hours of operation. After 102 hours of operation, the precipitation and the solution in the storage tank were separated. The precipitation in the storage tank was dissolved with hydrochloric acid for the measurement of the iron amount. The reactor was then washed with fresh basal medium to ensure that the pH value of the solution in the tank was ~6.8. In addition, three microbial samples were collected for the analysis of the microbial community distribution while steady-state achieved as long as 4 days under different oxygen concentrations (0%, 6% and 10% (v/v)).

### Analytical methods

The concentrations of ferrous ions and the total iron were measured by a modified 1,10-phenanthroline colorimetric method at 510 nm^41^. The concentration of ferric ions was determined by the difference between total iron and ferrous ions concentration. A liquid chromatography (Shimadzu, LC-20AT) was used for EDTA concentration measurement. The liquid chromatography was equipped with a Zorbax C8 column. The eluent consisted of 12.5% CH_3_OH, 0.26% tetrabutylammonium hydroxide, and 0.088% HCOOH. The retention time was 9 min. The inlet and outlet concentration of NO were measured via a chemiluminescent NO_*x*_ analyzer (Thermo, model 42*i*-HL). The X-ray photoelectron spectroscopy (XPS) measurements were performed on a RBD upgraded PHI-5000C ESCA system (Perkin-Elmer) with Mg Kα radiation (*hv* = 1253.6 eV), and binding energies were calibrated by using the containment carbon (C1s = 284.6 eV). The high-throughput sequencing (HTS) technology was used for genus classification. The details on the DNA extraction, PCR amplification, and sequencing can be found in the Supplementary Information. The sequencing data processing and analyses employed the Ribosomal Database Project (RDP), a classifier software based on Bergey’s taxonomy.

## Additional Information

**How to cite this article**: Li, W. *et al.* Pathway of FeEDTA transformation and its impact on performance of NO_x_ removal in a chemical absorption-biological reduction integrated process. *Sci. Rep.* **6**, 18876; doi: 10.1038/srep18876 (2016).

## Supplementary Material

Supplementary Information

## Figures and Tables

**Table 1:** Mass balance of iron during the operation of CABR integrated system.

iron inlet		iron outlet		deviation between iron inlet and outlet/mmol
inlet time/h	iron inlet amount/mmol	outlet pathway	iron outlet amount/mmol	
0	21.200	iron retained in the solution	17.99	
		iron precipitated in the storage tank	17.472	
72	18.350	iron loss due to the sampling	0.300	
total	39.550		35.762	3.788
