# Supplementary material for: Pathway of FeEDTA transformation and its impact on performance of NOx removal in a chemical absorption-biological reduction integrated process
Source: Sci Rep. 2016 Jan 8;6:18876. doi: 10.1038/srep18876 (PMC4705534; doi:10.1038/srep18876)
Supplement: Supplementary Information [file srep18876-s1.pdf]

**Appendix A. Supplementary Information:**

# Pathway of FeEDTA transformation and its impact on performance of $NO_x$ removal in a chemical absorption-biological reduction integrated process

Wei Li [1,2], Jingkai Zhao [1], Lei Zhang [1,3], Yinfeng Xia [1,2], Nan Liu [1], Sujing Li [1], Shihan Zhang [1,*]

[1] Key Laboratory of Biomass Chemical Engineering of Ministry of Education, Institute of Industrial Ecology and Environment, College of Chemical and Biological Engineering, Zhejiang University (Yuquan Campus), Hangzhou, 310027, China

[2] Institute of Environmental Engineering, Zhejiang University (Zijingang Campus), Hangzhou, 310058, China

[3] Zhejiang Industrial Environmental Protection Design & Research Institute Co., Ltd., Hangzhou, 310035, China

* Current address: Illinois State Geological Survey, University of Illinois at Urbana-Champaign, USA. Correspondence and requests for materials should be addressed to S.H. Z. (email: skyzhang@zju.edu.cn )

8 Pages

**Thermodynamic analysis of iron transformation without EDTA degradation**

For the solvent containing Fe(III)EDTA concentration of 10 mM at pH value of 6.4, the iron distribution was determined by the following reactions:

$$\mathrm{Fe(III)} + \mathrm{EDTA}^{4-} \leftrightarrow \mathrm{Fe(III)EDTA}^{-} \quad \text{(RS1)}$$

$$\mathrm{Fe(OH)_3} \leftrightarrow \mathrm{Fe}^{3+} + 3\mathrm{OH}^{-} \quad \text{(RS2)}$$

$K_f^{\theta'}$, the conditional stability constant of Fe(III)EDTA, defined as the chemical state of the complex, can be calculated by [1]:

$$K_f^{\theta'}\{\mathrm{Fe(III)EDTA}\}_1 = \frac{[\mathrm{Fe(III)EDTA}^{-}]_1}{[\mathrm{Fe}^{3+}]_1[\mathrm{EDTA}^{4-}]_1} \quad \text{(S1)}$$

$$\lg K_f^{\theta'} = \lg K_f^{\theta} - \lg\alpha_{Y(H)} - \lg\alpha_{M(OH)} \quad \text{(S2)}$$

$$[\mathrm{EDTA}^{4-}]_1 + [\mathrm{Fe(III)EDTA}^{-}]_1 = [\mathrm{Fe(III)EDTA}^{-}]_0 \quad \text{(S3)}$$

where $K_f^{\theta}$ is the stability constant under 298K and 100kPa; $\alpha_{Y(H)}$ is pH effect coefficient; $\alpha_{M(OH)}$ is hydroxyl effect coefficient; $[P]_1$ stands for the concentration of P, e.g, Fe(III)EDTA, $Fe^{3+}$, and $EDTA^{4-}$, M; $[Fe(III)EDTA^{-}]_0$ is the initial concentration of the Fe(III)EDTA.

The ion product $Q_{i,1}$ and the solubility product $K_{sp}$ of $Fe(OH)_3$ can be determined as:

$$Q_{i,1} = [\mathrm{Fe}^{3+}]_1[\mathrm{OH}^{-}]_1^3 \quad \text{(S4)}$$

$$K_{sp} = \exp\left(\frac{-\Delta G_r}{RT}\right) \quad \text{(S5)}$$

$$\Delta G_r = \Delta G(\mathrm{Fe}^{3+}) + 3\Delta G(\mathrm{OH}^{-}) - \Delta G(\mathrm{Fe(OH)_3}) \quad \text{(S6)}$$

where $\Delta G_r$ represents the Gibbs free energy change of the reaction (RS2) at T, $J \cdot mol^{-1}$; R is the universal gas constant; T is the Kelvin temperature, K; $\Delta G(\mathrm{P})$ stands for the Gibbs free energy of P at T, $J\ mol^{-1}$.

While reaction RS1 and RS2 reach equilibrium, the following equations are obtained.

$$Q_{i,1} = K\text{sp} \quad \text{(S7)}$$

$$\text{Fe(OH)}_3=([\text{EDTA}^{4-}]_1\text{-}[\text{Fe}^{3+}]_1)\text{V} \quad \text{(S8)}$$

Table S1 shows the parameters used for theoretical calculation of the iron precipitation as the form of $Fe(OH)_3$. Based on the above thermodynamic analysis, without considering the degradation of EDTA, 37% of the initial Fe(III) (14.8 mol iron) will be precipitated when the thermodynamic equilibrium is reached.

| **Original data** | | **Data got within calculation** | |
|---|---|---|---|
| $\lg K_f^{\theta}$ | 24.23 | $K_f^{\theta'}\{\text{Fe(III)EDTA}\}_1$ | $10^{14.47}$ |
| $\lg\alpha_{Y(H)}$ | 4.06 | | |
| $\alpha_{M(OH)}$ | $10^{5.7}$ | $\Delta G$ | 229027 J mol$^{-1}$ |
| T | 323 K | | |
| $\Delta G(Fe^{3+})$ | 3011 J mol$^{-1}$ | $K\text{sp}$ | $9.14\times10^{-38}$ |
| $\Delta G(OH^{-})$ | 156927 J mol$^{-1}$ | | |
| $\Delta G(Fe(OH)_3)$ | 696798 J mol$^{-1}$ | $[Fe^{3+}]_1$ | $9.14\times10^{-15.2}$ M |
| $[OH^{-}]_1$ | $10^{-7.6}$ M | | |
| V | 4 L | $[EDTA^{4-}]_1$ | $3.7\times10^{-3}$ M |

**Table S1.** Parameters used for thermodynamic analysis of iron transformation [1-3]

For the solvent containing both Fe(III)EDTA and Fe(II)EDTA at pH value of 6.8, since the solubility product of $Fe(OH)_3$ is twelve magnitude orders lower than that of $Fe(OH)_2$ [2], the $Fe^{3+}$ was precipitated prior to $Fe^{2+}$.

**DNA Extraction, PCR Amplification, and Quantitative Mixing**

DNA was extracted from 5ml supernatant of each biofilm sample using 3S DNA Isolation kit V2.2 for Environmental Samples (Biocolor, Shanghai). The extracted DNA samples were ascertained by electrophoresis in a 0.8% (w/v, g·mL$^{-1}$) agarose gel to assess the integrity. To identify the addition amount of DNA in PCR reaction, DNA was quantified by Qubit2.0 DNA Assay Kit.

PCR amplifications were carried out in a PTC-100TM Peltier Thermal Cycler (Bio-Rad Corp., USA) using extracted DNA as template. A pair of universal primers, 338F-GC (5'-GCCCGCCGCGCGCGGCGGGCGGGGCGGGGGCACGGGGGGACT CCTACGGGAGGCAGCAG-3') and 518R (5’-ATTACCGCGGCTGCTGG-3’), was used to amplify 16S rDNA. The PCR protocol included: 5 min denaturation at 94 ℃, 20 cycles of 94 ℃ for 1 min, 65-55 ℃ for 1 min (the annealing temperature decreased 1 ℃ every 2 cycles), 72 ℃ for 1 min, 10 cycles of 94 ℃ for 1 min, 55 ℃ for 1 min, 72 ℃ for 1 min, followed by 10 min at 72 ℃.

PCR products were electrophoresed in a 1.0% agarose gel, recycled by agarose gel extraction kit (cat: SK8131; Sangon Biotech, Shanghai). The recycled products were quantified by Qubit2.0 DNA Assay Kit. After all the DNA concentrations of recycled products were determined, the recycled products were mixed with a ratio of 1:1; then the mixture was used for the sequencing with Illumina MiSeq (Sangon Biotech Co., Ltd, Shanghai, China).

## Supplementary references


1. Jia, Z. S. *Inorganic and analytical chemistry*. 2nd edn, 262-272 (Higher Education Press, Beijing, 2008).
2. Haynes, W. M. *Handbook of Chemistry and Physics*. 95th edn, 8-1–8-104 (CRC Press, Boca Raton, 2014).
3. Ye, D. L. *Practical Handbook of thermodynamic data of inorganic materials*. 2nd edn, (Metallurgical Industry Press, Beijing, 2002).

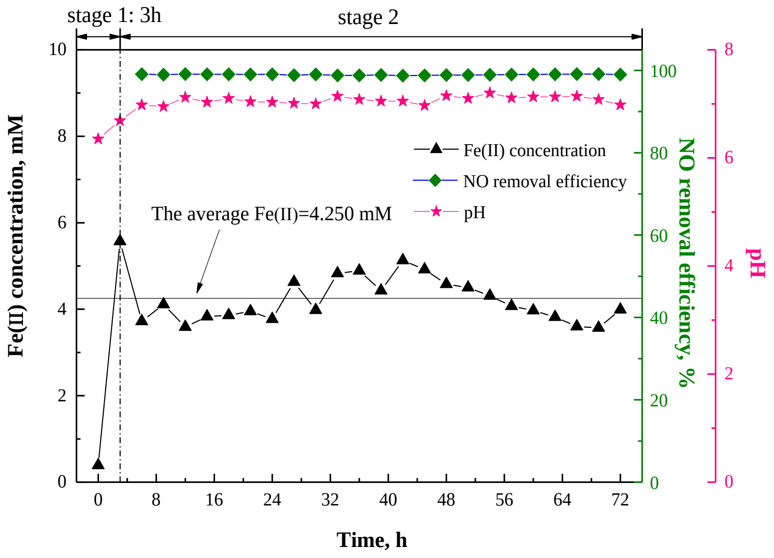


**Figure S1.** Continuous steady-state operation of the CABR system. (Stage 1: 0%(v/v) $O_2$, 0ppmNO, 15%(v/v) $CO_2$; Stage2: 6%(v/v) $O_2$, 400ppmNO, 15%(v/v) $CO_2$).

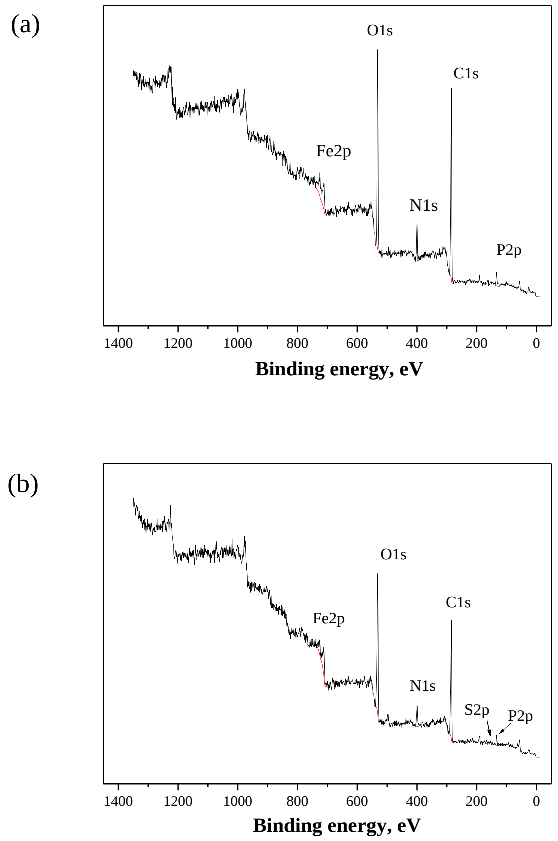


**Figure S2.** XPS survey spectrum of the samples (a) S-D and (b) S-B.

| Sample | Fe (2p) | | O (1s) | | N (1s) | | C (1s) | | P (2p) | | S (2p) | |
|---|---|---|---|---|---|---|---|---|---|---|---|---|
| | at% | BE(eV) | at% | BE(eV) | at% | BE(eV) | at% | BE(eV) | at% | BE(eV) | at% | BE(eV) |
| S-D | 2.57 | 712.49 | 24.58 | 531.8 | 7.48 | 400.05 | 60.79 | 285.18 | 4.58 | 133.37 | - | - |
| S-B | 2.47 | 711.78 | 28.91 | 531.44 | 6.44 | 399.56 | 56.23 | 285.2 | 4.32 | 133.24 | 1.64 | 170.58 |

**Table S1.** XPS data of the S-D and S-B samples
